# A New Functional Model for Prediction of Chaperone Activity of the Recombinant *M. tb* Acr (*α*-Crystallin) Using Insulin as Substrate

**DOI:** 10.1155/2019/2532045

**Published:** 2019-02-10

**Authors:** Gautam Krishnan, Utpal Roy

**Affiliations:** Department of Biological Sciences, BITS Pilani-K.K. Birla Goa Campus, NH 17B Bypass, Zuari Nagar, Goa 403726, India

## Abstract

*Mycobacterium tuberculosis* Acr is an important protein expressed in latent tuberculosis which is active as an oligomer in preventing misfolding of cellular proteins. In this study, *Mycobacterium alpha crystallin* (acr) gene was cloned and expressed in *Escherichia coli (E. coli)*. The recombinant Acr protein was purified by Nickel-NTA resin. The oligomeric state of Acr was confirmed by gel filtration chromatography using Sephacryl S-200 and Native-PAGE. Studies of chaperone activity were performed with insulin as a substrate at different mole ratios of Acr with 2 types of samples, His tag elutes (H) and His tag elutes with gel filtration (G). It was observed that the ratio of different sizes of oligomers (9 to 24 mers) had a significant effect on chaperone activity. Using the mole ratio of Acr for both (H) and (G) samples to insulin B chain and ratio of oligomers, we determined the number of Acr molecules binding to insulin as a model substrate. We found that if 1.5% of the insulin B chains are covered completely by the (G) samples, aggregation is completely inhibited as compared to 6% with (H) samples. Pre-heat treatment studies were carried out at 37°C, 60°C, and 70°C. Far-ultraviolet Circular Dichroism (UV-CD) analysis provided fresh insights into the role of *β*-sheets and *α*-helices in chaperone activity, particularly in (H) samples suggesting a reversible conformational transition from helices to sheets. This enabled us to formulate a functional model for binding of Acr to insulin B chains which incorporated 4 types of secondary structure molecules. This might be a useful tool for analyzing *in vitro* preparations of recombinant Acr and build more consensuses on the structure-activity relationship especially in terms of oligomeric ratios.

## 1. Introduction

*Mycobacterium tuberculosis* was originally a soil actinomycete that now infects human hosts, causing tuberculosis, one of the oldest diseases known to mankind, first reported, 4000 years ago [1]. In one out of every three persons, *M. tuberculosis* remains in a latent state, residing inside the macrophage. During this period, it accumulates large amounts of Hsp 16. Hsp 16 belongs to the family of *α*-crystallin as it has a carboxy (C)-terminal domain homologous to *α*-crystallin that can function as a molecular chaperone. Hence, it is called Acr under the domain of Rv2031c. Mycobacterial heat shock protein, Hsp 16, plays an important role in prevention of misfolding of proteins in hypoxic stress conditions [2]. It accumulates upto 25% of the total protein in bacteria and can be detected when the disease is in the latent form, where a program of genes is activated under the control of the DosR Regulon [3]. The mechanism of action of Acr in preventing thermal aggregation of substrates such as citrate synthase and catalase and preventing dithiothreitol-(DTT-) induced B chain aggregation of insulin and bovine serum albumin (BSA) has been studied [4–8]. These studies altogether provided insights into the mechanism of action of Acr. It is broadly agreed upon that the protein forms a trimer of trimers or a dodecamer and prevents misfolding of proteins [3, 8–10]. The exact mechanism of action of Acr in terms of oligomeric association and dissociation has given insights into the prevention of protein aggregation [11, 12].

These reports reveal dissociation and association of 7 to 9 oligomeric units for the recombinant Acr protein, using urea as a denaturant. It is known to form a nonamer and dodecamer that associates and dissociates, to function as a chaperone, preventing misfolding of denatured proteins in the hypoxic condition. It is also a heat shock protein known to have thermostable *β*-sheets. However, there are no significant reports on the functional role played by the proportion of oligomers in determining chaperone activity and also there is no common parameter to predict chaperone activity. Besides this, the reports are rather scanty on the consolidated secondary structure analysis of Far-UV CD profiles or its correlation to chaperone activity. The aim of this study was to gain insights into the mechanism of action of Acr in terms of binding to a model substrate, insulin B chain, and derives fresh insights into structure and activity relationships. The model we propose gives a mechanistic representation that can be extrapolated to help understand the phenomenon of latent tuberculosis, in terms of interaction of Acr with other proteins while the bacterium is in the dormant form. Here, based on activity, Far-UV CD study, and analysis of number of molecules that bind to the substrate, we present a new mechanism of binding of Acr using insulin as a substrate. A formula has been developed for chaperone activity of Acr, considering the oligomeric status of the same. Activity of Acr was expressed as a function of numerous parameters based on the data obtained and can be used to validate *in vitro* recombinant preparations.

## 2. Materials and Methods

### 2.1. Cloning Strategy

The approach was “Directional Cloning” of the polymerase chain reaction amplified mycobacterial acr gene into an Isopropyl thiogalactopyranoside (IPTG) inducible expression vector pET28a (Novagen), so that the 6-histidine tag would be added to the amino terminus. The primers were designed using the *M. tb* sequence in the TB database entry Rv2031c-hsp-X (http://www.tbdb.org), with NdeI in the forward primer and XhoI in the reverse primer (underlined). The primers designed were for the full-length gene as follows:

forward primer acr pET28 NdeI—5′ GGAATTCCATATGGCCACCACCCTTCCC 3′.

reverse primer acr pET28 XhoI—5′ CCGCTCGAGTCAGTTGGTGGACCGGATTCT 3′.

### 2.2. PCR Amplification of *M. tuberculosis* acr Gene and Preparation of Acr for Ligation

The gene of interest was amplified by diluting template DNA to 60 ng/*µ*l. A mixture of 120 ng of genomic DNA, 60 pmol of primer (synthesized by Integrated DNA Technology), and 2X EmeraldAmpGT PCR master mix (Takara) was made up to a final volume of 20 *µ*l. The PCR was set up in the thermal cycler in the following order: initial denaturation (94°C, 1.5 min), 25 cycles of denaturation (94°C, 1 min), annealing (61°C, 45 sec), extension (72°C, 45 sec), and final extension (72°C, 7 min). The PCR product was gel purified from a low melt agarose gel (Affymetrix) by using a gene clean kit (Macharay Nagel). 1 *µ*g of acr DNA was double digested with 50 units of NdeI and XhoI (Takara) using 10X buffer H for 20 hrs at 37°C. The digested DNA was then gene cleaned and used for ligation.

### 2.3. Vector Preparation and Ligation Reaction

Following the double digestion and dephosphorylation treatment, the vector was isolated and purified. After the overnight ligation reaction using the appropriate amount of treated vector and 50–100 ng of the insert DNA, the recombinant constructs were screened by colony PCR and confirmed by double digestion and release of the insert.

### 2.4. Expression and Purification Studies

Expression of the two acr containing clones, #3 and #6, was initially carried out in 50 ml of *E*. *coli* BL21DE3 cells, using 1 mM IPTG induction at 37°C for 3 hrs. Aliquots of 4 ml of uninduced and induced cultures were spun down at 8,000 g at 4°C for 10 mins. The cell pellets obtained were resuspended in 200 *µ*l 6X sodium dodecyl sulphate (SDS) loading dye and then boiled at 85°C and loaded on SDS-polyacrylamide gel electrophoresis (PAGE) 15% to check for expression of protein. The rest of the sample was sonicated using 10 mM Tris pH 7.0/5% glycerol followed by centrifugation at 20,000 g at 4°C for 30 mins. Aliquots of supernatant and pellet obtained were loaded on SDS-PAGE 15% to check for localization of protein. Scale-up expression studies of the recombinant Acr were done using a freshly streaked plate of the clone. For the optimization of expression and purification, varying volumes of secondary culture ranging from 50 ml to 1.8 L were used.

### 2.5. Purification of Acr Using Nickel-NTA Agarose (1.5 L Scale)

The acr Clone #3 was inoculated into 15 ml of LB kanamycin, subcultured into 1.5 L, and then induced with 0.5–1 mM IPTG at 0.4–0.6 OD_600_ for 3 hrs at 37°C. The cell pellet was lysed by sonicating in 60 ml lysis buffer containing 20 mM Tris pH 7.0, 500 mM NaCl, 5% glycerol, and 2X SigmaFast protease inhibitor (Catalogue no. S8830). The sonicate was centrifuged at 20,000 g for 30 mins at 4°C, and the supernatant obtained was bound to 3 ml of Nickel-NTA resin (HisPur™ Ni-NTA Resin Catalogue no. 88221, Thermo Scientific) for 2 hrs at 4°C, assuming a binding capacity of 20 mg/ml. The column was washed with 10 and 100 mM imidazole in a buffer containing 20 mM Tris pH 7.0, 500 mM NaCl, and 5% glycerol. The protein was eluted with a 3-step gradient of 300 mM, 400 mM, and 500 mM imidazole in a buffer containing 20 mM Tris pH 7.0, 300 mM NaCl, and 5% glycerol.

### 2.6. Identity of Expressed Protein by Matrix-Assisted Laser Desorption/Ionisation-Time of Flight (MALDI-TOF)

The Nickel-NTA purified protein was eluted out from the SDS-PAGE gel and subjected to tryptic digestion to confirm identity of the protein by mass spectrometry (MS/MS). Another band was cut out and subjected to molecular mass analysis by MALDI-TOF to confirm the mass of the protein. This analysis was done at Sandor Lifesciences, Hyderabad, India.

### 2.7. Gel Filtration of Acr

The Nickel-NTA eluted fractions containing protein (H) were dialyzed against 20 mM Tris pH 7.0, 100 mM NaCl, and 5% glycerol. 1.5 ml of the dialyzed protein, equivalent to 4 mg, was loaded twice on Sephacryl-200 Hiprep XK prepacked 16/70 column (120 ml) at a flow rate of 0.5 ml/min and buffer passed for a total of 1.5 column volumes using the AKTA Purifier System at C-CAMP, Bengaluru, India. The column was equilibrated using the same buffer and calibrated using Bio-Rad standards (Catalogue no. 151–1901) ranging in size from 1.5 kDa to 670 kDa.

### 2.8. Native-PAGE Analysis

An 8–16% Native-PAGE gel was used to run the (H) and (G) sample along with a control of bovine serum albumin (BSA) to check the size of oligomers present.

### 2.9. Assay Details

Insulin (Insugen R Injection, Biocon, India) at 2 different concentrations of 118 and 167 *µ*M was used as the substrate in a reaction volume of 0.25 ml with 20 mM Tris pH 7.0, 100 mM NaCl, and 5% glycerol. Aggregation was induced by the addition of 25 mM dithiothreitol (DTT) at 37°C and 60°C, and aggregation was measured over 30 mins by monitoring the increase in absorbance at 360 nm.

### 2.10. Dose-Dependent Studies

The inhibition of aggregation was measured after addition of Acr at varying concentrations ranging from 5 to 35 *µ*M for the (H) sample and 2 to 12 *µ*M for the (G) sample. The % inhibition was measured as follows:

(1) $$\begin{array}{c} \%\text{ inhibition}=100-\left[\frac{\text{final }{\text{OD}}_{360}\text{ of aggregated substrate with Acr}}{\text{final }{\text{OD}}_{360}\text{ of aggregated substrate without Acr}}\right]\times 100. \end{array}$$

A consolidated curve of % inhibition versus concentration of Acr and % inhibition versus mole ratio of Acr to insulin has been plotted for both (H) and (G) samples. The assay data figures constructed from the assay data have been replotted using the mole ratio of Acr to insulin versus % inhibition for both (H) and (G) samples at 37°C and 60°C. A consolidated curve of the mole ratio of Acr to insulin versus % inhibition has been plotted with overlay of all samples. Best fit polynomials are plotted for *R* ^2^ values greater than 0.95. A consolidated curve of the mole ratio of Acr to insulin versus % inhibition has been plotted with overlay of all samples. Best fit polynomials are plotted for *R* ^2^ values greater than 0.95.

### 2.11. Heat Treatment Studies

The heat treatment assays were carried out as follows: the (H) sample was assayed with insulin at a concentration of 11 *µ*M without pre-heat treatment and with 15 mins pre-heat treatment at 37°C, 60°C, and 70°C. For the (G) sample, assays were carried out at 1 *µ*M without pre-heat treatment and with 15 mins pre-heat treatment at 37°C, 60°C, and 70°C.

### 2.12. Circular Dichroism (CD) Analysis

Far-UV CD analysis of both (H) and (G) samples was carried out at protein concentrations of 0.5 mg/ml and 0.6 mg/ml, respectively, at the Centre for DNA Fingerprinting and Diagnostics (CDFD), Hyderabad, India, in the range of 190 to 260 nm using a Jasco spectropolarimeter and 1 mm cuvette with samples prepared in 10 mM sodium phosphate pH 7.0. The (H) samples were analyzed at 25°C and 37°C. The same samples were analyzed after the pre-heat treatment for 15 mins, at 37°C, 60°C, and 70°C. The (G) samples were analyzed at 37°C. The same samples were analyzed after pre-heating for 15 mins, at 37°C, 60°C, and 70°C.

### 2.13. Molecular Interaction Calculation

This was done using the website http://molbiol.edu.ru/eng/scripts/01_04.html, where the following assumptions were made: (i) reaction volume of 0.25 ml, (ii) insulin molecular weight of 3 kDa (assuming half of the molecule needs to be inhibited), and (iii) oligomeric ratio. The secondary structure calculations were done from the % of each secondary structure as a function of total number of molecules obtained from the CD data. The mass balance was calculated by totalling the number of molecules of the 4 types of secondary structures from the CD plots and assuming the oligomeric ratio obtained from Native-PAGE analysis, divided by the total theoretically expected, and this was expressed by the following formulae:

- number of molecules of Acr = amount in *µ*g *×* proportion of oligomers as per Native-PAGE/molecular weight and
- number of molecules of secondary structure of Acr = number of molecules of Acr at given temperature (with and without pre-heat treatment) *×* % of *β*-sheets/*α*-helices/random coils (from Far-UV CD data).

## 3. Results

### 3.1. Directional Cloning into NdeI and XhoI Site of pET28a

**Figure 1:**
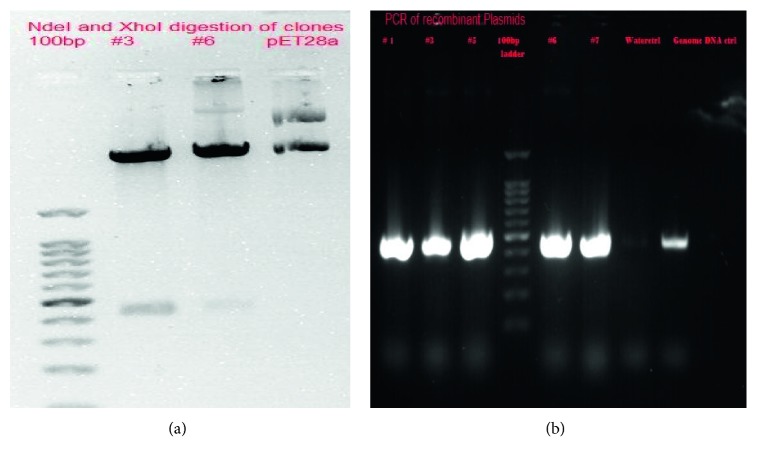
Cloning of *acr* gene. (a) Restriction digestion of clones: Lane 1: 100 bp ladder; Lane 2: Clone #3 digested with NdeI and XhoI; Lane 3: Clone #6 digested with NdeI and XhoI; Lane 4: pET28a control digested with NdeI and XhoI. (b) PCR of recombinant plasmids: Lanes 1 to 3: Clones #1, 3, and 5; Lane 4: 100 bp ladder; Lanes 5 and 6: Clones 6 and 7; Lane 7: negative control (water); Lane 8: positive control (genomic DNA).

The acr gene was amplified from the genome of *M. tuberculosis* H37Rv with an expected size of 434 base pairs. The PCR-amplified acr gene was cloned into the pET28a vector, and the construct was confirmed by restriction digestion and PCR of the plasmids (Figures 1(a) and 1(b)).

### 3.2. Expression and Nickel-NTA Purification

**Figure 2:**
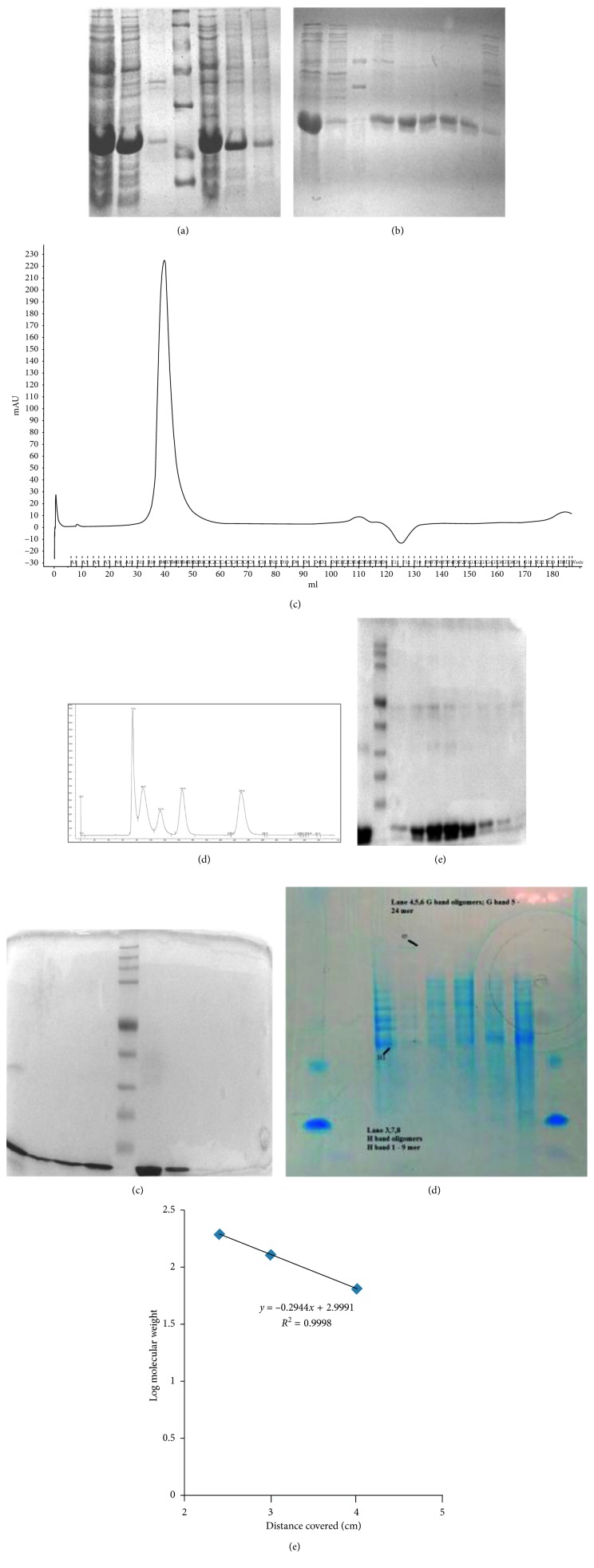
Expression and purification of acr-pET28a. (a) Expression of acr-pET28a #3 and #6: Lane 1: whole lysate #3; Lane 2: sonicate supernatant #3; Lane 3: sonicate pellet #3; Lane 4: markers 97 kDa, 66 kDa, 43 kDa, 30 kDa, 21 kDa, and 14 kDa; Lane 5: whole lysate #6; Lane 6: sonicate supernatant #6; Lane 7: sonicate pellet #6. (b) Nickel-NTA purification of acr-pET28a: Lane 1: load; Lane 2: flow through; Lane 3: markers 3, 6, 14, 21, 30, and 43 kDa; Lane 4: E1–E3; Lane 5: E5; Lane 6: E6; Lane 7: E7; Lane 8: E8; Lane 9: wash 1 + wash 2 elution gradients E1-E2 (300 mM imidazole), E3 (400 mM imidazole), and E5–E8 (500 mM imidazole). (c) Gel filtration run 2 chromatogram: *X* axis: UV _280_ nm; *Y* axis: elution time (min). (d) Gel filtration Bio-Rad Standards chromatogram: *X* Axis: UV _280_ nm; *Y* Axis: elution time (min); A, aggregates + thyroglobulin 670 kDa, 36.5 ml (73 mins); B, globulin 158 kDa, 44 ml (88 mins); C, ovalbumin 44 kDa, 55 ml (110 mins); D, myoglobin 17 kDa, 77 ml (154 mins); E, vitamin B12 1.5 kDa, 115 ml (230 mins). (e) 15% reducing SDS-PAGE analysis of gel filtration run 1: Lane 1: load; Lane 2: markers 315, 238, 171, 124, 70, 51, 42, 32, 26, and 10 kDa; Lanes 3–10: B4, B6, B7, B8, B9, B10, B11, and B12. (f)15% reducing SDS-PAGE analysis of gel filtration run 2: Lane 1: load; Lanes 2–4: B4, B5, and B6; Lane 5: markers 315, 238, 171, 124, 70, 51, 42, 32, 26, and 10 kDa; Lane 6: B7 and B8; Lane 7: B9; Lane 8: B11; Lane 9: B10. (g) Native-PAGE of his tag elute (H) and gel filtration elute (G): Lanes 1 and 9: BSA control; Lane 3: (H); Lanes 4, 5, and 6: (G); Lanes 7 and 8: (H). (h) Plot of log molecular weight of the different forms of BSA in Native-PAGE. The different sizes of BSA 66, 132, and 198 kDa were plotted against distance migrated and a log mol weight was plotted in MS excel, and the equation was displayed and used to calculate the oligomer size of (H) and (G) samples.

The 50 ml culture showed expression of Acr in *E. coli* BL21DE3 upon 1 mM IPTG induction. Two clones labeled #3 and #6 were selected for expression. Clone #3 showed higher protein expression than Clone #6 and was used for all further studies (Figure 2(a)). The expression level was found in the range of 50–60 mg/l of the recombinant protein. The Acr protein ran closer to the 21 kDa marker, a little higher than the expected 18 kDa. The soluble protein bound to the Nickel-NTA column was eluted by the 3-step (300, 400, and 500 mM) imidazole gradient. The 500 mM imidazole fraction showed approximately 95% purity as revealed by SDS-PAGE (Figure 2(b)).

### 3.3. Gel Filtration Purification

Gel filtration experiments showed the Acr peaks appearing in the void volume between the size of 158 kDa and 670 kDa (relative to the standards), suggesting its oligomeric nature. In a representative of 8 runs, a major peak was observed at 37.5 ml (75 min) while the peak ended at 44 ml (88 min), the position corresponding to the globulin peak of 158 kDa (Figure 2(c)). The Sephacryl S200 Hi Prep 16/70 column was calibrated with Bio-Rad standards (Catalogue no. 151–1901); all the peaks appeared as per the expected pattern aggregates with thyroglobulin 670 kDa, globulin 158 kDa, ovalbumin 44 kDa, myoglobin 17 kDa, and vitamin B12 1.5 kDa (Figure 2(d)). A 15% reducing SDS-PAGE was run to analyze the peaks obtained in the gel filtration runs (Figures 2(e) and 2(f)). The peak 1 of both the runs showed Acr along with an upper band in the position of 38–40 kDa which could possibly be a dimeric form which appeared in other gel filtration runs (data not shown). No protein was observed in the other 2 peaks of both the runs indicating that the Acr protein exists predominantly in an oligomeric form. A mixture of oligomers was observed in the (G) sample which was confirmed by Native-PAGE analysis.

### 3.4. Sequencing

The protein sequence was confirmed as Acr, with both molecular mass determination and 9 peptide fragment sequences found matching with Acr sequence using Mascot software. The molecular mass was found to be 18.3 kDa corresponding to the cloned sequence 16 kDa plus an additional 2.1 kDa from the amino terminal histidine tag (Supplementary Figures A1, A2).

### 3.5. Native-PAGE Analysis

The nondenaturing 8–16% gradient gel showed a mixture of 9 mers to 12 mers in case of (H) samples and 9 mers to 24 mers in case of (G) samples. The ratio of 9 mers was higher in the (H) samples compared to the (G) samples, where the 24 mers ratio was higher, and it appeared as a prominent band, running higher than all the other bands (Figure 2(g)). A plot of log molecular weight versus distance migrated was used to estimate the size and ratio of the oligomers present (Figure 2(h)). An assumption of ±0.05 to 0.1 cm was considered in the migration distances. BSA ran as monomer, dimer, and trimer (66, 132, and 198 kDa). By analyzing the relative intensity of the bands, a proportion of each of the oligomeric size was estimated ranging from 15 and 25 to 40% (Table 1). The proportion of the bands was verified by using ImageJ software with a variation of ±10%.

### 3.6. Activity of (H) versus (G) Samples

**Figure 3:**
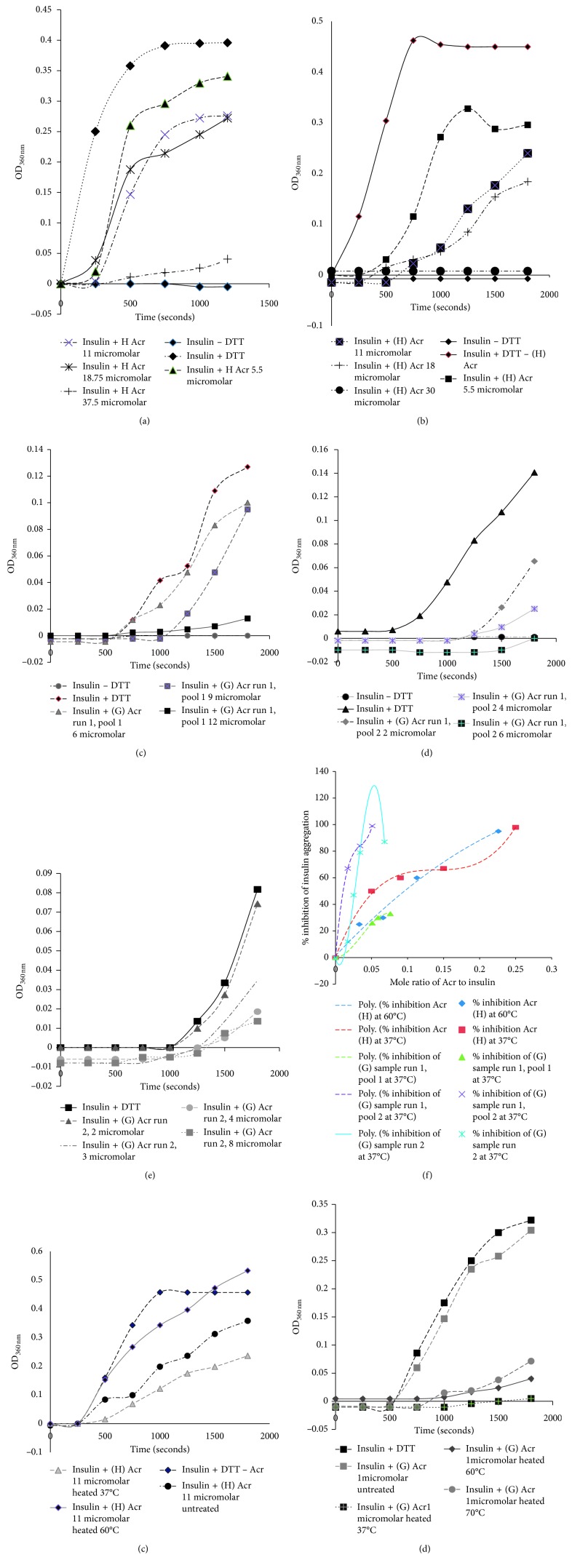
Chaperone activity of (H) and (G) samples at 37°C and 60°C. (a) Chaperone activity of (H) at 60°C: insulin (1 mg/ml) was used for the assay and aggregation measured at 360 nm over 20 mins by adding 25 (mM DTT). Acr was used at 4 different concentrations: 5.5 *μ*M, 11 *μ*M, 18.75 *μ*M, and 37.5 *μ*M. (b) Chaperone activity of (H) at 37°C: insulin (1 mg/ml) was used for the assay, and aggregation was measured at 360 nm over 30 mins by adding 25 mM DTT. Acr was used at 4 different concentrations of 5.5, 11, 18, and 30 *μ*Μ. (c) Chaperone activity of (G) pool 1: run 1: insulin 1 (mg/ml) was used for the assay and aggregation measured at 360 nm over 30 mins by adding 25 mM DTT. Acr was used at concentrations of 6, 9, and 12 *μ*M. (d) Chaperone activity of (G) pool 2, run 1: insulin (1 mg/ml) was used for the assay and aggregation measured at 360 nm over 30 mins by adding 25 mM DTT. Acr was used at concentrations of 2, 4, and 6 *μ*M. (e) Chaperone activity of (G) run 2: insulin (1 mg/ml) was used for the assay and aggregation measured at 360 nm over 30 mins by adding 25 mM DTT. Acr was used at concentrations of 2, 3, 4, and 8 *μ*M. (f) Consolidated curve of Figures 3(a) to 3(e): the mole ratios of Acr to insulin obtained from Figures 3(a) to 3(e) (Table 2) were replotted versus % inhibition and numerical best fits with polynomials plotted. (g) Chaperone activity of (H) at 37°C after pre-heat treatment at 37°C and 60°C: insulin (1 mg/ml) was used for the assay and aggregation measured at 360 nm over 30 mins by adding 25 mM DTT. Acr was used at a concentration of 11 *μ*M, with no pre-heat treatment and with pre-heat treatment at 37°C and 60°C. (h) Pre-heat treatment studies and chaperone activity of (G): insulin (1 mg/ml) was used to induce aggregation with 25 mM DTT and aggregation measured at 360 nm over 30 mins. The concentration of Acr used was 1 *μ*M, and pre-heat treatment was carried out at 37°C, 60°C, and 70°C for 15 minutes.

(H) samples showed similar activity at 37°C and 60°C with the only difference being in the nature of the polynomial best fits obtained (Figures 3(a) and 3(b)). Remaining assays have been performed at 37°C. Pre-heat treatment of (H) samples led to improvement of activity at 37°C but no improvement was observed at 60°C (Figure 3(g)) and 70°C (data not shown). This observation was analyzed subsequently by Far-UV CD studies. However, (G) samples showed improvement in activity after the pre-heat treatments at 37°C, 60°C, and 70°C (Figure 3(h)). The (G) samples of 3 different pools of 2 runs showed better activity than (H) samples. The concentration of 12 *µ*M of (G) samples was required to achieve 95% inhibition as compared to 30 *µ*M of (H) samples. The consolidated curve used to compare activity of all the (H) and (G) samples showed interesting patterns (Figure 3(f)). The (H) sample showed a shift of the best fit to a 3rd order from a 2nd order, whereas the (G) samples showed a shift of the polynomial best fits to the left. The reasons for these shifts are explained in the latter section with the help of CD data. Nevertheless, the observations indicate that the higher ratio of oligomers contribute to better chaperone activity (Table 2).

### 3.7. Far-UV CD Studies

**Figure 4:**
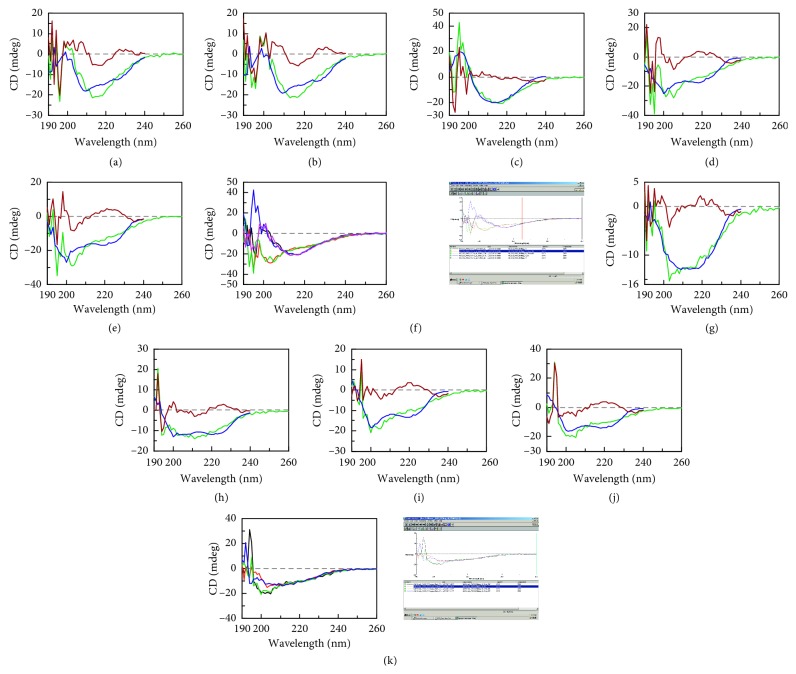
CD of (H) samples: (H) samples were checked using the Jasco spectropolarimeter (Far-UV CD) 190 nm to 260 nm at 25°C and 37°C (without pre-heat treatment) and 37°C, 60°C and 70°C (with pre-heat treatment for 15 mins). (a) Far-UV CD analysis of (H) samples at 25°C without pre-heat treatment. (b) Far-UV CD analysis of (H) samples at 37°C without pre-heat treatment. (c) Far-UV CD analysis of (H) samples after pre-heat treatment for 15 mins at 37°C. (d) Far-UV CD analysis of (H) samples after pre-heat treatment for 15 mins at 60°C. (e) Far-UV CD analysis of (H) samples after pre-heat treatment for 15 mins at 70°C. (f) Overlay of Far-UV CD analysis of (H) samples: 25°C without pre-heat treatment (indicated in black); 37°C without pre-heat treatment (indicated in purple); 37°C with pre-heat treatment (indicated in blue); 60°C with pre-heat treatment (indicated in green); and 70°C with pre-heat treatment (indicated in red). (g) Far-UV CD analysis of (G) samples at 37°C without pre-heat treatment. (h) Far-UV CD analysis of (G) samples at 37°C with pre-heat treatment. (i) Far-UV CD analysis of (G) samples with pre-heat treatment at 60°C. (j) Far-UV CD analysis of (G) samples with pre-heat treatment at 70°C. (k) Overlay of Far-UV CD analysis of (G) samples: 37°C without pre-heat treatment (indicated in red); 37°C with pre-heat treatment (indicated in blue); 60°C with pre-heat treatment (indicated in green); 70°C with pre-heat treatment (indicated in brown).

Far-UV CD analysis revealed that the (H) and (G) samples showed different proportions of *α*-helices, *β*-sheets, and random coils (Figure 4; Tables 3 and 4). For the (H) samples, the *β*-sheets increased from **11%** to **30–62%**, after pre-heat treatment. The (H) samples without pre-heat treatment showed higher fraction of *α*-helices (**22–26%**) which decreased to **3.4–14%** after pre-heat treatment. For (G) samples, the *β*-sheets changed from **45%** to **25–55%** after pre-heat treatment. The fraction of *α*-helices was **9.6%** and changed to **3.9–14%** after pre-heat treatment. The fraction of random coils was found to be **34.4%** and increased to **40–46%** following the pre-heat treatment.

### 3.8. Heat Treatment Studies

The (G) samples, after pre-heat treatment at 37°C, showed complete inhibition at 1 *μ*M at a mole ratio as low as 0.0084 (Figure 3(h)). On the other hand, the (G) samples after pre-heat treatment at 60°C and 70°C showed **94%** and **85%** inhibition, respectively (Figure 3(h)). Upon pre-heat treatment at 37°C, (H) samples at 11 *µ*M showed slight improvement in activity while at 60°C, it showed a decline in activity (Figure 3(g)) and at 70°C, it showed similar result as that at 60°C (data not shown).

### 3.9. Molecular-Level Binding of Acr to Insulin B Chains

Mathematical calculations revealed that, at 12 *µ*M, the (G) sample showed approximately 95% inhibition of 118 *µ*M insulin. If we correlate this observation with the Native-PAGE data of the (G) sample, 54 *µ*g (a total of 21.6 *µ*g of the 16 mers and 8 *µ*g of the 9, 10, 11 and 12 mers each), in terms of number of molecules, for a reaction volume of 0.25 ml, this works out to 3.01 × 10^13^, 2.98 × 10^13^, 2.78 × 10^13^, 2.48 × 10^13^, 2.28 × 10^13^ molecules, or, a total of 1.35 × 10^14^ molecules. An assumption of ±10% was considered for all these calculations based on the approximation of migration distance and proportion of bands as described in Results section of Native-PAGE analysis. Number of molecules corresponds to a total of 1.5% of the total molecules of insulin (8 × 10^15^) assuming half of the 6 kDa B chain is the target to be inhibited. By covering 1.5% of the B chain of insulin, 95% inhibition was achieved. The same method was used to calculate the number of molecules of Acr that bound to insulin in the study of (G) samples after pre-heat treatment. Before pre-heat treatment, the number of molecules of Acr of the 1 *μ*M sample was 2.49 × 10^12^ molecules of 24 mers, 2.5 × 10^12^ molecules of 9 mers, 2.28 × 10^12^ molecules of 10 mers, 2.05 × 10^12^ molecules of 11 mers, and 1.88 × 10^12^ molecules of 12 mers, or a total of 1.13 × 10^13^ molecules. This merely covered 0.13% of the total molecules of the B chain. However, when pre-heat treated at 37°C, 60°C, and 70°C, the chaperone activity improved significantly, showing similar inhibition as if the number of molecules was 13-fold higher. The results are summarized in Table 5. In the case of (H) samples, 30 *µ*M was required to achieve 95% inhibition (135 *µ*g) which corresponds to a total of 94.5 *μ*g of the 9 mers, 13.5 *μ*g each of the 10 mers, 11 mers, and the 12 mers. In terms of number of molecules, this corresponds to 3.5 × 10^14^ molecules of 9 mers, 4.38 × 10^13^ molecules of 10 mers, 4.125 × 10^13^ molecules of 11 mers, and 3.763 × 10^13^ molecules of 12 mers, or a total of 4.73 × 10^14^ molecules. This corresponds to a total of 6% of the total number of molecules of insulin B chain. The results are summarized in Table 5.

### 3.10. Molecular-Level Binding of Acr to Insulin B Chain in terms of Secondary Structure

**Figure 5:**
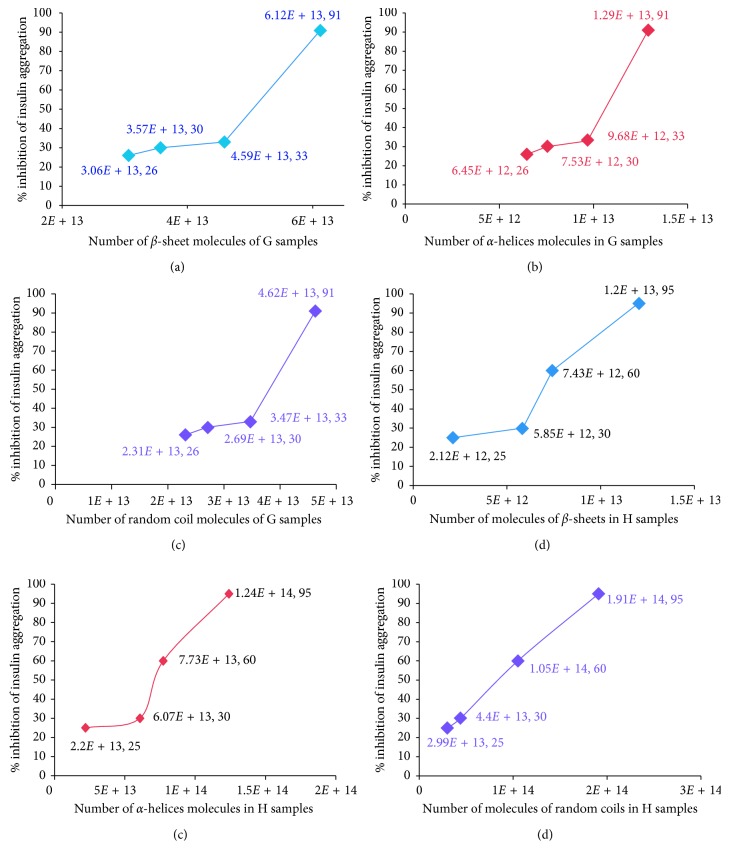
Molecular interaction of (G) and (H) samples of Acr without pre-heat treatments. (a) Plot of molecules of *β*-sheets versus % inhibition for (G) samples without pre-heat treatment: the % of *β*-sheets (45.5%) obtained from the CD analysis (Table 4) was multiplied by the total number of Acr molecules at 4 different concentrations of Acr 6, 7, 9, and 12 *μ*M to estimate the actual number of *β*-sheets interacting with insulin. The total amount of Acr in the reaction was recalculated based on the Native-PAGE data and the ratio of oligomers (40% of 24 mers and 15% of 9, 10, 11, and 12 mers) (Table 1). This calculation was repeated for all (G) samples. (b) Plot of *α*-helices versus % inhibition for (G) samples without pre-heat treatment: the % of *α*-helices for (G) samples was 9.6% (Table 4), and this was multiplied with the 4 different concentrations of Acr 6, 7, 9, and 12 *μ*M to estimate the actual number of *α*-helices interacting with insulin. (c) Plot of random coils versus % inhibition for (G) samples without pre-heat treatment: the % of random coils of (G) samples (34.4%) (Table 4) was multiplied with 4 different concentrations of Acr 6, 7, 9, and 12 *μ*M to estimate the actual number of random coils interacting with insulin. The total amount of Acr in the reaction was recalculated based on the Native-PAGE data and the ratio of oligomers (40% of 24 mers and 15% of 9, 10, 11, and 12 mers) (Table 1). This calculation was repeated for all (G) samples. (d) Plot of molecules of *β*-sheets versus % inhibition for (H) samples: the % of *β*-sheets (2.5%) (Table 3) was multiplied by the total number of Acr molecules at 4 different concentrations of Acr 5.5, 11, 18, and 30 *μ*M to estimate the actual number of *β*-sheets interacting with insulin. The total amount of Acr in the reaction was recalculated based on Native-PAGE data and the ratio of oligomers (70% of 9 mers and 10% of 10, 11, and 12 mers) (Table 1). This process was repeated for all (H) samples. (e) Plot of *α*-helices versus % inhibition for (H) samples without pre-heat treatment: the % of *α*-helices for the (H) samples was 26% (Table 3), and this was multiplied with the 4 different concentrations of Acr 5.5, 11, 18, and 30 *μ*M to estimate the actual number of *α*-helices interacting with insulin. The total amount of Acr in the reaction was recalculated based on Native-PAGE data and the ratio of oligomers (70% of 9 mers and 10% of 10, 11, and 12 mers) (Table 1). This process was repeated for all (H) samples. (f) Plot of random coils versus % inhibition for (H) samples without pre-heat treatment: the % of random coils for the (H) samples was 36.2% (Table 3), and this was multiplied with 4 different concentrations of Acr 5.5, 11, 18, and 30 *μ*M to estimate the actual number of turns interacting with insulin. The total amount of Acr in the reaction was recalculated based on Native-PAGE data and the ratio of oligomers (70% of 9 mers and 10% of 10, 11, and 12 mers) (Table 1). This process was repeated for all (H) samples.

The plots of *β*-sheets versus % inhibition for (G) samples revealed an interesting pattern, and 6-7 × 10^13^ molecules were required for complete inhibition of insulin (Figure 5(a)). Graphs are also plotted for (G) samples in terms of *α*-helices and random coils (Figures 5(b) and 5(c)). The number of molecules of *α*-helices bound to insulin was greater than 1.4 × 10^13^. The number of molecules of random coils that bound to insulin was greater than 5 × 10^13^. Graphs of secondary structure analyses for (H) samples have been plotted (Figures 5(d)–5(f)) in order to explain activity and pre-heat treatment results. The number of *β*-sheets required for complete inhibition was 1.195 × 10^13^ molecules. A similar analysis was carried out for *α*-helices and random coils, which showed an interesting pattern. For *α*-helices of (H) samples, it required about 1.2 × 10^14^ molecules to completely inhibit insulin B chain aggregation. The number of random coils required to ensure 95% inhibition of insulin was 1 × 10^14^. The following observations are reported in terms of number of secondary structure molecules required to completely inhibit insulin aggregation before pre-heat treatment (Table 6); the amount of *β*-sheets required in (G) samples was 5.5 times more than in (H) samples whereas the amount of *α*-helices was 10 times less than in (H) samples without pre-heat treatment. The amount of random coils in (G) samples was 4 times less as compared to (H) samples. The mass balance obtained showed **106%** for (H) samples and **91%** for (G) samples (Table 6). This is the ratio of total molecules obtained from secondary structure data and the molecules theoretically expected from Native-PAGE analysis, based on the oligomer ratio.

### 3.11. Molecular-Level Binding of Acr (with Pre-Heat Treatment) to Insulin B Chain in terms of Secondary Structure

For (G) samples, for 1 *μ*M Acr with pre-heat treatment, 2.8–7 × 10^12^ molecules of *β*-sheets, 4.4 × 10^11^ – 1.6 × 10^12^ molecules of *α*-helices, and 4.56–5.3 × 10^12^ molecules of random coils were sufficient for complete inhibition (Supplementary data A3, A4, A5). In this case, a clear trend of reduction of activity was recorded with concomitant reduction of random coils. Pre-heat treatment of (H) samples at 11 *μ*M, at 37°C, showed that 1 × 10^14^ molecules of *β*-sheets, 2.44 × 10^13^ molecules of *α*-helices, and 3.268 × 10^13^ molecules of random coils were required for **48%** inhibition of the insulin B chain aggregation after pre-heat treatment (Supplementary data A6, A7, A8).

## 4. Discussion

We successfully cloned the PCR-amplified acr gene using the directional cloning strategy and overexpressed the Acr protein in the soluble form. This enabled us to carry out several functional and characterization studies. In gel filtration chromatography, Acr appeared as an oligomer, comparing with Bio-Rad standards. Earlier reports revealed Acr to be a nonameric 158 kDa in the form of trimer of trimers [4], 210 kDa [10], and 193 kDa [8, 9]. The Native-PAGE gel analysis indicated a clear difference in the pattern of oligomers with 9 mers dominant in the (H) samples as compared to the (G) samples where the 24 mers band was predominant. The mixture of 9 to 24 mers in the (G) sample indicated an association of the oligomers after gel filtration that influences the chaperone activity. Previously, the process of dissociation and association was extensively studied using urea with a wild type and a mutant Acr [11]. They reported that it requires mild denaturant (4M) urea to find the intermediates before oligomerization. Reports have also shown the importance of the nonamer as a prerequisite to dissociate in order to exhibit chaperone activity; the generally accepted mechanism is a trimer of trimers aggregating to form the nonamer [4] for unleashing the chaperone activity. We observed varying amounts of 9 mers to 12 mers in the (H) samples and 9 mers to 24 mers in the (G) samples. We could infer that dissociation of a higher molecular weight oligomer improves chaperone activity. The 3-fold higher activity of (G) samples compared to (H) samples could be explained by the proportion of oligomers.

### 4.1. Analysis from Mole Ratio Graphs

The polynomial best fit plotted in Figure 3(f) indicates interesting trends. The shift of the best fits to the left indicates better binding to insulin. When more data points are added from more batches of *in vitro* preparations of Acr, a hypothesis could be built based on data of (H) and (G) samples. The Native-PAGE analysis of the (G) samples indicated a higher proportion of Acr oligomers and commensurately the increased chaperone activity. This could also be a function of the concentration of the soluble Acr protein and the dynamic association and dissociation of different amounts of 9 mers to 24 mers. Later batches of Acr preparation showed 6 and 8 mers in (H) samples that were not found in (G) samples and showed better activity for (G) samples as compared to (H) samples (data not shown). A detailed investigation in finding the correlation between the 2^nd^ and 3^rd^ order coefficients in the polynomial curves needs to be carried out in order to understand how the nature of oligomers might affect the type of polynomial graphs.

### 4.2. Far-UV CD Data

This was generated under the same conditions at which the activity assays were carried out. The nature of inhibition of the (H) sample at 37°C showed a change compared to that at 60°C, which indicating that, at lower concentrations of Acr, the inhibition of aggregation at 37°C is double at mole ratios of 0.03 to 0.10. However, at higher mole ratios of 0.115 to 0.25, the inhibition was similar. This could be a reflection of the higher amount of *β*-sheets formed at 60°C (**48.5%**). However, the reduced amount of *α*-helices could have played a role here, as they are reduced to **3.7%** from **26%** when pre-heated at 60°C but this is compensated by higher % of *β*-sheets. This is an important observation that needs further investigation and currently indicates a shift in conformation from *α*-helices to *β*-sheets upon exposure to pre-heat treatment. Whether a similar phenomenon occurs with Acr *in vivo* is a matter of investigation. The (G) samples at 37°C, without pre-heat treatment, showed improved activity as compared to the (H) sample at 37°C, without pre-heat treatment. The fraction of *β*-sheets in the (G) samples was estimated to be **45%** as compared to **2.5%** in the (H) samples. The (G) samples showed better activity even though the amount of *α*-helices (**9.6%**) recorded was less in the (H) samples (**26%**) (Tables 3 and 4).

### 4.3. Molecular-Level Binding of Acr to Insulin B Chain in terms of Secondary Structure

Based on the observations (Table 1; Figures 5(a) to 5(f); Supplementary data A3 to A8), we are tempted to propose a theoretical and preliminary model of the secondary structure of Acr based on number of molecules of the different conformations: *β*-sheets, *α*-helices, and random coils. The underlying assumption for this model is the ratio of oligomers, rather than a single oligomeric size.

### 4.4. Heat Treatment Studies

Heating of (G) samples resulted in improved binding which might be due to transition of random coils into *β*-sheets and thereby increasing the effective hydrophobic surfaces of oligomeric Acr (Figure 3(h)). These results suggest an optimum range of **25%** to **45%** of *β*-sheets for manifesting the chaperone activity, warranting further study to determine the effect of pre-heat treatment on availability of surfaces for insulin binding. It was reported that mild heating of *M. tb* Acr at 30°C increased hydrophobic surfaces [5], and it was proposed that, for human *α*-crystallin, hydrophobic binding sites are provided by *β*-sheets in the oligomer and also by coassociation with the C-terminal of a monomer [13]. The improvement in activity at 37°C for the (H) sample could be due to an increase in *β*-sheets from **2.5** to **62%**, which corroborates the activity profile obtained wherein the best fit graphs changed from 37°C to 60°C (Figures 3(a), 3(b), 3(f), and 3(g); Table 2). The (H) sample, at 60°C and 70°C, initially showed an inhibition that was higher than the unheated sample, but the increased OD values in the latter part indicated either dissociation or unstable nature of *β*-sheets (Figure 3(g)). It can be conjectured that a mixture of *β*-sheets, *α*-helices, and random coils might have contributed to chaperone activity with number of *β*-sheets playing a significant role in (G) samples, due to the higher molecular weight oligomer.

### 4.5. Comparison with Existing Studies

**Figure 6:**
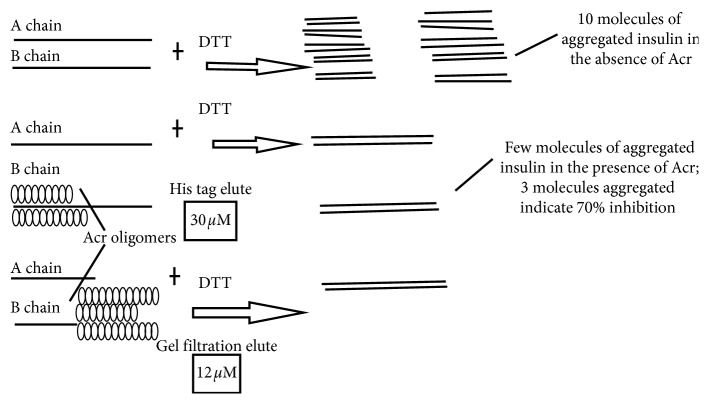
Schematic presentation of binding of Acr to insulin. A pictorial schematic of binding of Acr to insulin B chain was drawn to simulate the molecular interaction of protein and substrate.

It has been reported that the Far-UV CD spectrum of *M. tb* Acr, at 25°C, exhibited a minimum of 217 nm, indicating a possibility of high number of *β*-sheets. The denaturation studies using 1 M and 2 M guanidine hydrochloride exhibited disorder in the secondary structure [5]. It was reported that Fourier-transform infrared (FTIR) spectroscopy at 25°C exhibited predominance in *β*-sheets (67%). This is one of the two reports that provided a complete breakup of all the different conformations, ranging from *α*-helices to turns in Acr. The studies evidenced that structural changes were more from 25°C to 40°C than from 40°C to 60°C and the disorder increased from 8 to 10% due to the increase in random coils. The same study also predicted that the highly thermostable sheets (67%) help in chaperone activity of Acr [6]. We report a decrease in *β*-sheets from 11.5 to 2.5% for (H) samples on heating from 25°C to 37°C and then an increase to **62.9%** upon pre-heat treatment at 37°C for 15 mins which enhances the chaperone activity (Figure 3(g)). Contrary to this, pre-heat treatment at 60°C and 70°C led to decrease in chaperone activity in (H) samples. It can be conjectured that the reduction in *β*-sheets from 62.9% to 48.5% at 60°C and to 30% at 70°C could have contributed to this phenomenon. On the other hand, the (G) samples, at 37°C, without pre-heat treatment, exhibited 45% of *β*-sheets which is much higher than the (H) samples. The higher ratio of 24 mers that was observed in the Native-PAGE analysis could have contributed to this observation. Gel filtration could be another factor that might have potentially contributed to the higher number of *β*-sheets in (G) samples. The primary eluate in gel filtration appeared in the void suggesting a range of molecular weights from 158 kDa to 670 kDa, thus enhancing the possibility of two to three 24 mers molecules associating to enhance chaperone activity. Upon pre-heat treatment, *β*-sheets decreased from 45% to 25% and then increased to 51% and 54% at 60°C and 70°C, respectively. However, a decrease in activity was observed; this could be a function of the low concentration of the sample used (1 *μ*M) and needs to be investigated further at different doses to draw further conclusions on patterns of structure and activity. Secondary structure variations using Far-UV CD from 25°C to 65°C were recorded, and *β*-sheets were found to be dominant in Acr [9]. Another study at the same temperature range revealed the presence of decreased *β*-sheets and increased random coils. The same report showed a shift in Far-UV CD spectra towards the left [14]. In the present study, heat-treated (G) samples demonstrated an increase in random coils upon heating (Table 4, Figures 4(h)–4(j)). Also, there was a decrease in *β*-sheets from 5 × 10^12^ to 2.85 × 10^12^ on pre-heat treatment at 37°C. However, on pre-heat treatment at 60°C and 70°C, the *β*-sheets increased to 5.8 × 10^12^ and 6.2 × 10^12^, respectively. It can be conjectured therefore that stronger association of higher molecular weight oligomers might have contributed to this. A separate study demonstrated Far-UV CD spectra of Acr at 25°C. This revealed a predominance of *β*-sheets [15]. Far-UV CD analysis at different temperatures from 37°C to 60°C have shown an alteration in the secondary structure with a shift towards the left of the Far-UV CD spectra [16]. Similar observations were recorded in the current study for the (G) samples (Figure 4(k)). Far-UV CD studies of 34% *β*-sheets and 16% *α*-helices at 25°C have also been recorded. They reported the breakup of different features of the secondary structure [8]. In brief, we recorded Far-UV CD values of (G) samples with the predominance of *β*-sheets (25 to 45%) in (G) samples and the increase in random coils to **40%** upon pre-heating that are in agreement with existing reports. Our results for (H) samples indicate a bigger role for *α*-helices in chaperone activity and a weak and transient formation of *β*-sheets that dissociates upon heating to 37°C, 60°C, and 70°C. This transition phenomenon highlights the important role of gel filtration as a method to obtain enhanced chaperone activity. We have compared our CD data with the predicted secondary structure analysis using PSIPRED and found that the (G) samples match more closely with the theoretical amount of *β*-sheets and *α*-helices (Table 7). This clearly supports the idea of a transitory *α* helix in the (H) sample which gets converted to *β*-sheets after gel filtration chromatography. This has been reported for other proteins, especially ovalbumin [17]. We too have similar observations for both (H) and (G) samples (Table 7). A schematic diagram (Figure 6) can explain the difference in activity between (H) and (G) samples (as shown below). All the above results can be partially explained by the Native-PAGE data of both (H) and (G) samples, and we propose the following model for binding of recombinant Acr to insulin (Figure 6). The diagram explains the molecular level interaction of Acr with insulin B chain with the two representative concentrations of (H) and (G) of 12 and 30 *μ*M, respectively. The diagram also explains that higher amount of oligomers probably helps in improved coverage of the insulin B chains.

### 4.6. Proposed Formulae: Chaperone Activity of *M. tb* Acr on Insulin

(2) $$\begin{array}{c} A\;\;=\;\;f\left\{R_{1},\;\;\ldots ,\;\;R_{n},\;\;M,\;\;C,\;\;\beta ,\;\;\alpha ,\;\;R_{\text{C}},\Delta \right\}, \end{array}$$

where *A* = activity is defined as >95% inhibition of DTT-induced aggregation of insulin B chain; *f* = function of; *R* = ratio of oligomers in the sample (R^1–9^ mers, R^2–10^ mers, R^3–11^ mers, R^4–12^ mers, and R^5–24^ mers); *M* = number of molecules of Acr, bound to 3 kDa of insulin; *C* = % of insulin B chain covered; *β* = molecules of *β*-sheets in the sample, where 5-6 × 10^13^< *β* < 1-2 × 10^14^; *α* = molecules of *α*-helices in the sample, where 10^13^ < *α* < 10^14^; *R*_c_ = molecules of random coils, where 5 × 10^13^ < *R*_c_ < 2 × 10^14^; Δ = heating of samples, where 37°C < Δ < 70°C.

The values given in the proposed formula have been calculated after consolidation of activity, CD spectroscopy, and molecular interaction data obtained for both (H) and (G) samples. Each functional parameter is presented as a range of values which would vary as per the molar concentrations of Acr and insulin. This might serve as a useful molecular tool to analyze *in vitro* samples of Acr for the structure-activity relationship.

## 5. Conclusions

We have proposed a molecular tool to estimate the % of molecules of insulin covered that enables us to predict the chaperone activity. We have analyzed the chaperone activity of soluble *in vitro* Acr in terms of ratio of oligomers present that can predict with near precision the extent to which binding to the insulin B chain may occur. Thus, a 2- to 3-fold difference in the chaperone activity was determined. We also used the mole ratio of Acr to insulin for all analyses, a parameter that has been rarely used in all the previous reports on Acr. A consolidated Far-UV CD secondary structure analysis with both (H) and (G) samples and pre-heat treatment studies at three different temperatures explained the variations in the chaperone activity. The transition from *α*-helices to *β*-sheets in the (H) and (G) samples is a new finding. The abundance of oligomers might accelerate the prevention of malfolding of protein substrates, within the macrophage. This happens inside the host, especially, when the *M. tb* is in the latent form inside the macrophage in a hypoxic condition. The new functional model of analyzing *in vitro* soluble recombinant Acr preparations proposed here can add valuable insights into its mechanism of action *in vivo.*

## Figures and Tables

**Table 1:** Oligomer ratio calculation: oligomer sizes are calculated, using BSA monomer, dimer, and trimer as a reference (66, 132, and 198 kDa), and the log of molecular weight plotted versus the distance travelled in centimeter. From this calculation, molecular weights are extrapolated for the different bands viewed in (H) (bands 1, 2, 3, and 4) and (G) samples (bands 1, 2, 3, 4, and 5) using the equation used in Figure 2(h) (*y* = 0.294*x* + 2.999). The proportion of bands was estimated using ImageJ software. For distance migrated, the error was ±0.05 cm, and for estimating the proportion, an assumption of ±10% was made.

BSA (mol. wt.)	Log mol wt.	Distance (cm)			
198	2.29	2.4 ± 0.24			
132	2.12	3 ± 0.03		Monomer	
66	1.82	4 ± 0.14		18	
			Size (kDa)	Average oligomer size	% Oligomer fraction of total amount of protein
(H) band 1	2.21 ± 0.04	2.7 ± 0.05	162 ± 15	8.84	70 ± 7
(H) band 2	2.264 ± 0.04	2.5 ± 0.05	184.93 ± 15	10	10 ± 1
(H) band 3	2.29 ± 0.03	2.35 ± 0.05	204 ± 18	11.16	10 ± 1
(H) band 4	2.35 ± 0.02	2.2 ± 0.05	229.8 ± 18	12.48	10 ± 1
				Ratio of 10 mers to 12 mers	30 ± 3
				9 mers	70 ± 7
(G) band 1		Same as (H)		9	15 ± 1.5
(G) band 2				10	15 ± 1.5
(G) band 3				11	15 ± 1.5
(G) band 4				12.48	15 ± 1.5
(G) band 5	2.7	1.2	436	24	40 ± 4
				Ratio of 10 mers to 24 mers	85 ± 8.5
				9 mers	15 ± 1.5

**Table 2:** Chaperone activity of (H) and (G) versus mole ratio at 60°C and 37°C. Polynomial best fit has been plotted in MS excel with XY scatter function in charts along with trendline with equation and *R* ^2^ value.

Mole ratio of Acr (H) sample to insulin at 60°C	% inhibition
0	0
0.033	25
0.066	30
0.113	60
0.226	95
Mole ratio of Acr (H) sample to insulin at 37°C	% inhibition
0	0
0.05	50
0.09	60
0.15	67
0.25	98
Mole ratio of Acr (G) sample, run 1, pool 1 to insulin at 37°C	% inhibition
0	0
0.051	26
0.059	30
0.076	33
0.102	91
Mole ratio of Acr (G) sample, run 1, pool 2 to insulin at 37°C	% inhibition
0	0
0.017	67
0.034	84
0.051	99
Mole ratio of Acr (G) sample, run 2 to insulin at 37°C	% inhibition
0	0
0.017	12
0.025	47
0.034	79
0.068	87

**Table 3:** Consolidated CD raw data of proportion of molecules of secondary structure of (H) samples.

CD of (H) samples at 25°C	% of total
*α*-helices	22.7
*β*-sheets	11.5
Turns	30.3
Random coils	35.5
CD of (H) samples at 37°C	% of total
*α*-helices	26.0
*β*-sheets	2.5
Turns	35.3
Random coils	36.2
CD of (H) samples at 37°C with pre-heat treatment	% of total
*α*-helices	14.0
*β*-sheets	62.9
Turns	4.3
Random coils	18.8
CD of (H) samples at 60°C with pre-heat treatment	% of total
*α*-helices	3.7
*β*-sheets	48.5
Turns	7.9
Random coils	39.9
CD of (H) samples at 70°C with pre-heat treatment	% of total
*α*-helices	8.4
*β*-sheets	30.0
Turns	15.4
Random coils	46.1

**Table 4:** Consolidated CD raw data of proportion of molecules of secondary structure of (G) samples.

CD of (G) samples at 37°C without pre-heat treatment	% of total
*α*-helices	9.6
*β*-sheets	45.5
Turns	10.5
Random coils	34.4
CD of (G) samples at 37°C with pre-heat treatment	% of total
*α*-helices	14.3
*β*-sheets	25.0
Turns	14.0
Random coils	46.6
CD of (G) samples at 60°C with pre-heat treatment	% of total
*α*-helices	3.9
*β*-sheets	51.1
Turns	2.9
Random coils	42.1
CD of (G) samples at 70°C with pre-heat treatment	% of total
*α*-helices	5.4
*β*-sheets	54.6
Turns	0.0
Random coils	40.0

**Table 5:** Molecular interaction studies. Calculations of molecular level interaction of Acr (G), (H), and (G) with pre-heat treatment (concentrations of 12 *μ*M, 30 *μ*M, and 1 *μ*M, respectively) are based on the following assumptions: reaction volume 0.25 ml, insulin molecular weight of B chain 3 kDa, and Acr molecular weight 18 kDa. The total amount of Acr in the reaction is recalculated based on the Native-PAGE data and the ratio of oligomers assuming an error estimation of ±10 % (Table 1).

	Mol. wt. (kDa)	Amount (*µ*g)	Molecules of insulin B chain alone	% of insulin B chain covered
Insulin	6	174	8.73 × 10^15^	
Acr	18			
(G) (12 *µ*M)	18	54	1.8 × 10^15^	
40% of 24 mers	288	21.6	3.011 × 10^13^ ± 0.301 × 10^13^	
15% of 9 mers	162	8.1	2.98 × 10^13^ ± 0.298 × 10^13^	
15% of 10 mers	180	8.1	2.7 × 10^13^ ± 0.27 × 10^13^	
15% of 11 mers	198	8.1	2.48 × 10^13^ ± 0.248 × 10^13^	
15% of 12 mers	216	8.1	2.28 × 10^13^ ± 0.228 × 10^13^	
Total Acr molecules			1.35 × 10^14^	1.54
(H) (30 *µ*M)		135	4.52 × 10^15^ ± 0.452 × 10^15^	
70% of 9 mers	162	94.5	3.5 × 10^14^ ± 0.35 × 10^14^	
10% of 10 mers	180	13.5	4.39 × 10^13^ ± 0.439 × 10^13^	
10% of 11 mers	198	13.5	4.13 × 10^13^ ± 0.413 × 10^13^	
10% of 12 mers	216	13.5	3.76 × 10^13^ ± 0.376 × 10^13^	
Total Acr molecules			4.73 × 10^14^ ± 0.473 × 10^14^	6
(G) (1 *µ*M) with pre-heat treatment	18	4.5	1.50 × 10^14^	
40% of 24 mers	288	1.8	2.49 × 10^12^ ± 0.249 × 10^12^	
15% of 9 mers	162	0.68	2.53 × 10^12^ ± 0.253 × 10^12^	
15% of 10 mers	180	0.68	2.28 × 10^12^ ± 0.228 × 10^12^	
15% of 11 mers	198	0.68	2.07 × 10^12^ ± 0.207 × 10^12^	
15% of 12 mers	216	0.68	1.88 × 10^12^ ± 0.188 × 10^12^	
Total Acr molecules			1.13 × 10^13^	0.13

**Table 6:** A comparison of (H) and (G) samples in terms of 4 secondary structures to achieve 95% inhibition of insulin aggregation. The number of molecules of 4 secondary structures required to achieve 95% inhibition was compared for (H) and (G) samples and the total number of molecules (mass balance) compared with the theoretical number of molecules expected by Native-PAGE and the amount used for assay.

Secondary conformations and other parameters	Molecules in (H) samples	Molecules in (G) samples	Ratios (G)/(H)
*β*-sheets	1.1 × 10^13^	6.1 × 10^13^	5.55
*α*-helices	1.2 × 10^14^	1.29 × 10^13^	0.12
Random coils	1.9 × 10^14^	4.63 × 10^13^	0.08
Total molecules	4.81 × 10^14^	1.33 × 10^14^	0.28
Actual expected by Native-PAGE	4.54 × 10^14^	1.47 × 10^14^	0.32
Mass balance	106%	91%	—

**Table 7:** Comparison of secondary structures of (H) and (G) samples at 37°C from PSIPRED. The N-terminal sequence of Acr has been used to calculate the theoretical secondary structure followed by comparison with CD data of (H) and (G) samples as shown in Tables 2 and 3, respectively.

Amino acids (162)	*α*-Helices	*β*-Sheets	Coils and turns
Theoretical (by PSIPRED)	3%	36%	61%
(G) samples	9.6%	45.5%	44.9%
(H) samples	26%	2.5%	71.5%

## Data Availability

All data will be made available by the authors on request.

## Supplementary Materials

Supplementary Materials Supplementary data S1: Sequencing and mass confirmation of Acr A1: tryptic digest of recombinant Acr and matching of peptide fragments using Mascot. Supplementary data A2: molecular mass determination of recombinant Acr. Supplementary data S2: molecular interactions of secondary structure molecules of (H) and (G) samples after pre-heat treatments. Supplementary data A3: plot of *β*-sheets versus % inhibition for (G) samples (1 *μ*M) with pre-heat treatment. The % of *β*-sheets for 1 *μ*M Acr was calculated as 45.5% at 37°C without pre-heat treatment and 25.0, 51.1, and 54.6%, respectively, for 37°C, 60°C, and 70°C, respectively (Table 4) and multiplied by the number of molecules. Supplementary data A4: plot of *α*-helices versus % inhibition for (G) samples (1 *μ*M) with pre-heat treatments. The % of *α*-helices for 1 *μ*M Acr sample was calculated to be 9.6% for 37°C without pre-heat treatment and 14.3, 3.9, and 5.4% for 37°C, 60°C, and 70°C, respectively (Table 4), and multiplied by the number of molecules. Supplementary data A5: plot of random coils versus % inhibition for (G) samples (1 *μ*M) with pre-heat treatments. The % of random coils for 1 *μ*M Acr sample was calculated as 34.4% for 37°C without pre-heat treatment and 46, 40, and 42% for 37°C, 60°C, and 70°C, respectively (Table 4), and multiplied by the number of molecules. Supplementary data A6: plot of *β*-sheets for (H) samples (11 *μ*M) with pre-heat treatments. The % of *β*-sheets for 11 *μ*M of (H) samples was calculated as 2.5% for 37°C without pre-heat treatment and 62.5 and 48.5% for 37°C and 60°C, respectively (Table 3), and multiplied by the number of molecules. Supplementary data A7: plot of *α*-helices versus % inhibition for (H) samples (11 *μ*M) with pre-heat treatments. The % of *α*-helices for 11 *μ*M of (H) samples was calculated as 26% for 37°C without pre-heat treatment and 14 and 3.7% for 37°C and 60°C, respectively (Table 3), and multiplied by the number of molecules. Supplementary A8: plot of random coils versus % inhibition for (H) samples (11 *μ*M) with pre-heat treatments. The % of random coils for 11 *μ*M of (H) samples was calculated as 36% for 37°C without pre-heat treatment and 18.8 and 39.9% for 37°C and 60°C, respectively (Table 3), and multiplied by the number of molecules.
