# Supplementary figures and images for: A New Functional Model for Prediction of Chaperone Activity of the Recombinant M. tb Acr (α-Crystallin) Using Insulin as Substrate
Source: Can J Infect Dis Med Microbiol. 2019 Feb 10;2019:2532045. doi: 10.1155/2019/2532045 (PMC6387734; doi:10.1155/2019/2532045)

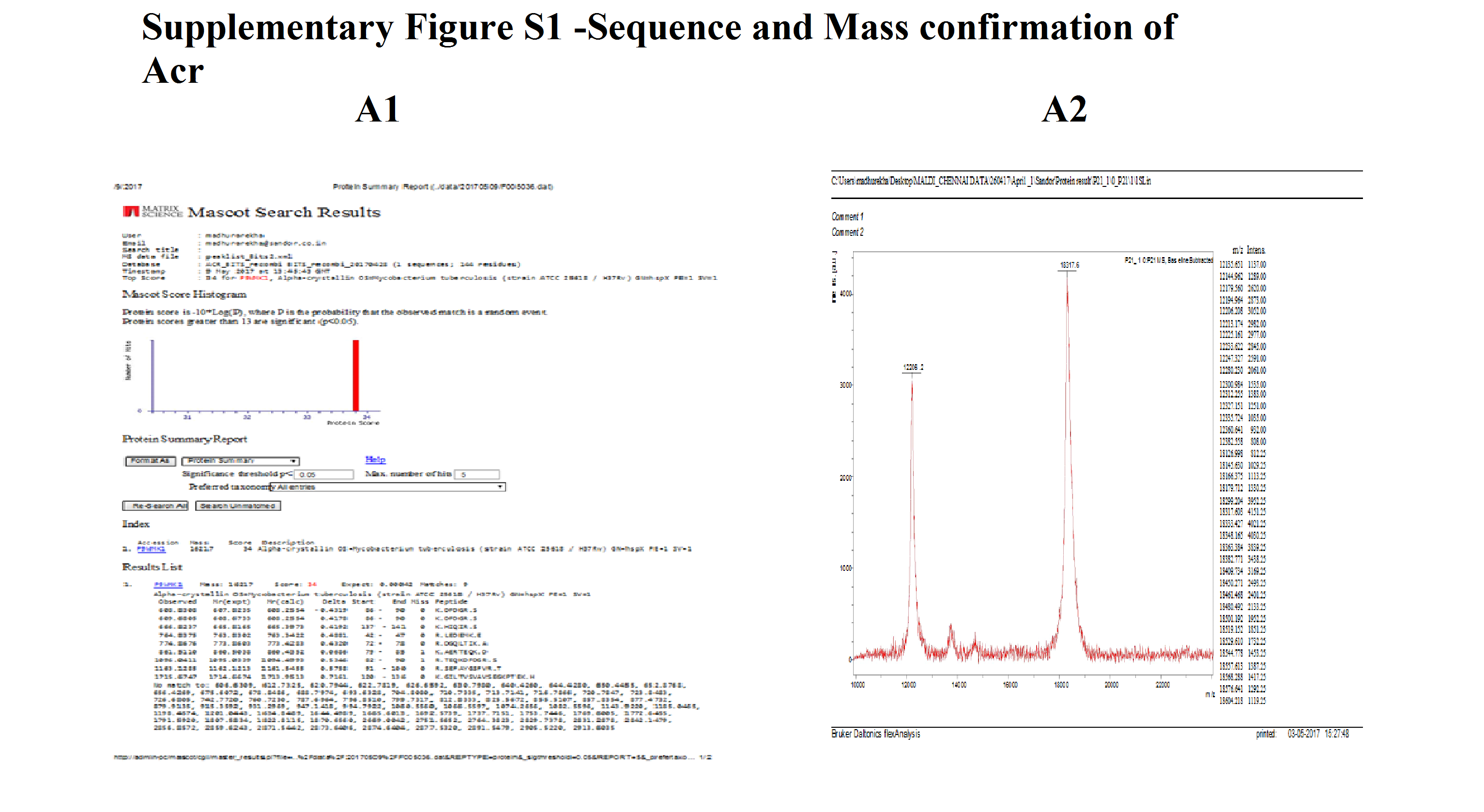

Supplement: Supplementary Materials — Supplementary data S1: Sequencing and mass confirmation of Acr A1: tryptic digest of recombinant Acr and matching of peptide fragments using Mascot. Supplementary data A2: molecular mass determination of recombinant Acr. Supplementary data S2: molecular interactions of secondary structure molecules of (H) and (G) samples after pre-heat treatments. Supplementary data A3: plot of β-sheets versus % inhibition for (G) samples (1 μM) with pre-heat treatment. The % of β-sheets for 1 μM Acr was calculated as 45.5% at 37°C without pre-heat treatment and 25.0, 51.1, and 54.6%, respectively, for 37°C, 60°C, and 70°C, respectively (Table 4) and multiplied by the number of molecules. Supplementary data A4: plot of α-helices versus % inhibition for (G) samples (1 μM) with pre-heat treatments. The % of α-helices for 1 μM Acr sample was calculated to be 9.6% for 37°C without pre-heat treatment and 14.3, 3.9, and 5.4% for 37°C, 60°C, and 70°C, respectively (Table 4), and multiplied by the number of molecules. Supplementary data A5: plot of random coils versus % inhibition for (G) samples (1 μM) with pre-heat treatments. The % of random coils for 1 μM Acr sample was calculated as 34.4% for 37°C without pre-heat treatment and 46, 40, and 42% for 37°C, 60°C, and 70°C, respectively (Table 4), and multiplied by the number of molecules. Supplementary data A6: plot of β-sheets for (H) samples (11 μM) with pre-heat treatments. The % of β-sheets for 11 μM of (H) samples was calculated as 2.5% for 37°C without pre-heat treatment and 62.5 and 48.5% for 37°C and 60°C, respectively (Table 3), and multiplied by the number of molecules. Supplementary data A7: plot of α-helices versus % inhibition for (H) samples (11 μM) with pre-heat treatments. The % of α-helices for 11 μM of (H) samples was calculated as 26% for 37°C without pre-heat treatment and 14 and 3.7% for 37°C and 60°C, respectively (Table 3), and multiplied by the number of molecules. Supplementary A8: plot of random coils versus % inh [file 2532045.f1.zip › 2532045.f1/S1(A1-A2) .tif]

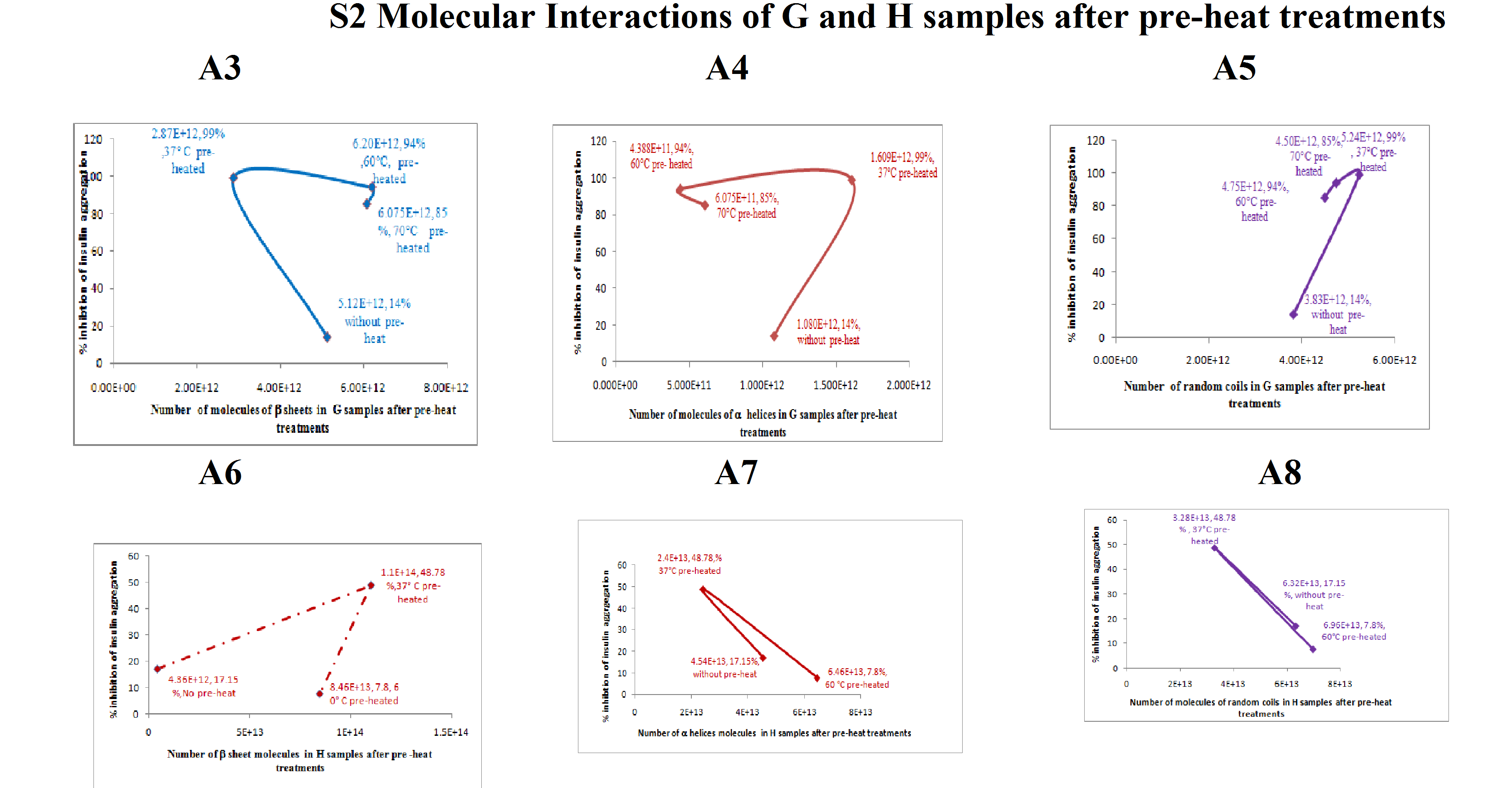

Supplement: Supplementary Materials — Supplementary data S1: Sequencing and mass confirmation of Acr A1: tryptic digest of recombinant Acr and matching of peptide fragments using Mascot. Supplementary data A2: molecular mass determination of recombinant Acr. Supplementary data S2: molecular interactions of secondary structure molecules of (H) and (G) samples after pre-heat treatments. Supplementary data A3: plot of β-sheets versus % inhibition for (G) samples (1 μM) with pre-heat treatment. The % of β-sheets for 1 μM Acr was calculated as 45.5% at 37°C without pre-heat treatment and 25.0, 51.1, and 54.6%, respectively, for 37°C, 60°C, and 70°C, respectively (Table 4) and multiplied by the number of molecules. Supplementary data A4: plot of α-helices versus % inhibition for (G) samples (1 μM) with pre-heat treatments. The % of α-helices for 1 μM Acr sample was calculated to be 9.6% for 37°C without pre-heat treatment and 14.3, 3.9, and 5.4% for 37°C, 60°C, and 70°C, respectively (Table 4), and multiplied by the number of molecules. Supplementary data A5: plot of random coils versus % inhibition for (G) samples (1 μM) with pre-heat treatments. The % of random coils for 1 μM Acr sample was calculated as 34.4% for 37°C without pre-heat treatment and 46, 40, and 42% for 37°C, 60°C, and 70°C, respectively (Table 4), and multiplied by the number of molecules. Supplementary data A6: plot of β-sheets for (H) samples (11 μM) with pre-heat treatments. The % of β-sheets for 11 μM of (H) samples was calculated as 2.5% for 37°C without pre-heat treatment and 62.5 and 48.5% for 37°C and 60°C, respectively (Table 3), and multiplied by the number of molecules. Supplementary data A7: plot of α-helices versus % inhibition for (H) samples (11 μM) with pre-heat treatments. The % of α-helices for 11 μM of (H) samples was calculated as 26% for 37°C without pre-heat treatment and 14 and 3.7% for 37°C and 60°C, respectively (Table 3), and multiplied by the number of molecules. Supplementary A8: plot of random coils versus % inh [file 2532045.f1.zip › 2532045.f1/S2(A3-A8) .tif]
